# Sensitivity of Genome-Wide-Association Signals to Phenotyping Strategy: The PROP-TAS2R38 Taste Association as a Benchmark

**DOI:** 10.1371/journal.pone.0027745

**Published:** 2011-11-23

**Authors:** Ulrich K. Genick, Zoltán Kutalik, Mirko Ledda, Maria C. Souza Destito, Milena M. Souza, Cintia A. Cirillo, Nicolas Godinot, Nathalie Martin, Edgard Morya, Koichi Sameshima, Sven Bergmann, Johannes le Coutre

**Affiliations:** 1 Department of Food-Consumer Interaction, Nestlé Research Center, Lausanne, Switzerland; 2 Department of Medical Genetics, University of Lausanne, Lausanne, Switzerland; 3 Swiss Institute of Bioinformatics, Lausanne, Switzerland; 4 Sensonomic Laboratory of Alberto Santos Dumont Research Support Association & IEP Sirio Libanes Hospital, São Paulo, Brazil; 5 Department of Radiology, Faculdade de Medicina, Universidade de São Paulo, São Paulo, Brazil; 6 Organization for Interdisciplinary Research Projects, The University of Tokyo, Yayoi, Bunkyo-ku, Tokyo, Japan; Duke University, United States of America

## Abstract

Natural genetic variation can have a pronounced influence on human taste perception, which in turn may influence food preference and dietary choice. Genome-wide association studies represent a powerful tool to understand this influence. To help optimize the design of future genome-wide-association studies on human taste perception we have used the well-known TAS2R38-PROP association as a tool to determine the relative power and efficiency of different phenotyping and data-analysis strategies. The results show that the choice of both data collection and data processing schemes can have a very substantial impact on the power to detect genotypic variation that affects chemosensory perception. Based on these results we provide practical guidelines for the design of future GWAS studies on chemosensory phenotypes. Moreover, in addition to the TAS2R38 gene past studies have implicated a number of other genetic loci to affect taste sensitivity to PROP and the related bitter compound PTC. None of these other locations showed genome-wide significant associations in our study. To facilitate further, target-gene driven, studies on PROP taste perception we provide the genome-wide list of p-values for all SNPs genotyped in the current study.

## Introduction

An identical taste stimulus may elicit substantially different perceptions in different individuals. Some individuals may detect the same stimulus at a lower concentration than others. And, at concentrations above the detection threshold, the perceived intensity of the stimulus may vary between individuals. This variation in taste sensitivity may affect food liking as well as food choice [1]–[6] and may ultimately impact health outcomes.

Variations in taste sensitivity are driven both by genetic and by environmental factors (*e.g.* dietary habits or childhood experiences). Traditionally the study of environmental factors has garnered much of the attention. But, with the help of high-throughput genotyping arrays and genome-wide-association studies (GWAS) [7] it is now possible to investigate the genetic contribution to variation in human chemosensory perception systematically [8], [9].

In the current study we investigate how the choice of phenotyping strategy influences the chances of success for such genome-wide association studies on chemosensory perception, with a particular focus on taste perception.

### Measuring chemosensory phenotypes

In comparison to most of the phenotypes targeted in medically-oriented GWASs (e.g. body height, weight, BMI, blood pressure etc.) chemosensory phenotypes are relatively difficult to measure precisely. While simple phenotyping approaches for chemosensory perception exist - for example subjects may simply be asked to rate the intensity of a single tastant solution on an intensity scale – these simple measurements are prone to be diluted by substantial measurement uncertainties. Potential sources of these uncertainties have been identified and increasingly powerful - but also increasingly complex - procedures to account for these uncertainties have been identified [10]–[12]. Other phenotyping methods that are independent of subjective ratings exist [13]. For example the staircase method [14] determines detection thresholds by testing, in blind taste tests, the subjects' ability to distinguish samples containing the compound of interest from reference samples. The greater accuracy expected from such objective methods comes with a substantial increase in experimental effort. Similarly, replicate measurements may help to improve the phenotypic data by averaging out day-to-day variations in taste sensitivity, but, again, come at the cost of increased experimental effort. Which of these different phenotyping options will perform best in a GWAS scenario?

Here we report on a study that uses the well-known genotype-phenotype association between the detection thresholds for the bitter compound PROP and a specific variation in the TAS2R38 bitter taste receptor (HGNC:9584) as a benchmark to compare the relative power of different taste phenotyping strategies for GWAS applications.

### Genetic variation in the TAS2R38 bitter receptor and perception of PROP

In 1932 Arthur Fox [15] reported a striking inter-subject variability in the perception of the bitter compound Phenylthiocarbamide (PTC). While some individuals, including Fox himself, were essentially unable to detect the compound's bitterness, others were much more sensitive. Those individuals who could perceive the compound even at low concentrations are often referred to as “tasters” while those who perceive the compound only at much higher concentrations are called “non-tasters”.

The availability of a simple test - placing a PTC-soaked paper-strip on subjects' tongues and recording if they perceived its bitter taste – allowed the taster phenotype to be determined quickly, on large panels and with reasonable accuracy. Based on these tests, it soon became clear that the ability to taste PTC at low concentrations was a genetic trait that was inherited in near-mendelian fashion [16].

Using classical linkage surveys the genetic variation underlying PTC sensitivity was initially mapped to a region on chromosome 7q3 [17], [18]. Later Dennis Drayna and colleagues [19] used linkage analysis and large-scale DNA sequencing to pinpoint the TAS2R38 bitter taste receptor gene as the site of the responsible genetic variations. Single nucleotide polymorphisms (SNPs) at basepairs 145, 785 and 886 (C->G, C->T and C->T) within the coding region of the TAS2R38 receptor gene lead to three amino-acid changes (proline->alanine, alanine->valine and valine-> isoleucine) at amino acids 49, 262 and 296 respectively [20]. As it turns out, these three variations in the receptor tend to be co-inherited such that for the vast majority of cases the TAS2R38 receptor exists either in the proline,alanine,valine taster version (PAV in the single letter amino acid code) or in the alanine,valine,isoleucine (AVI) non-taster version.

In more recent studies PTC is often replaced by propylthiouracil (PROP). PROP displays nearly the same sensory properties as PTC and, due to its status as an approved drug, PROP poses fewer safety concerns.

## Results

### Psychophysics Data

**Figure 1:**
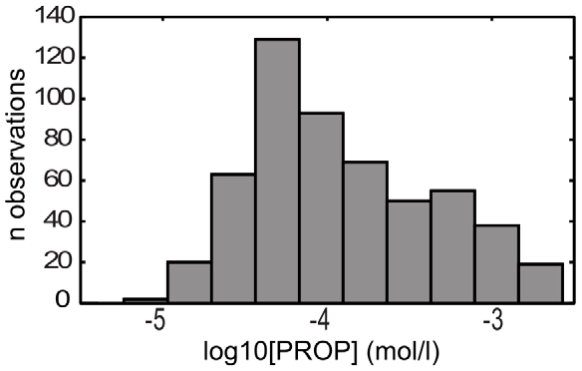
Inter-subject variability of PROP detection thresholds. Distribution of detection thresholds for the bitter compound PROP. Thresholds were measured by the staircase method. Concentrations are represented on a logarithmic scale.

To characterize the taste sensitivity of the study's subjects to PROP we determined both detection thresholds and supra-threshold intensity ratings for aqueous solutions of this compound. Figure 1 shows a histogram of the log-transformed detection thresholds with its familiar bi-modal distribution. The range of observed thresholds is comparable to those reported by others [13], [21], [22]. Replication of results on separate days allowed the determination of day-to-day, intra-subject variability. The mean difference of the two thresholds determined per subject (d  =  |log_10_[thresh1] – log_10_[thresh2]|) equals 0.32 log units.

**Figure 2:**
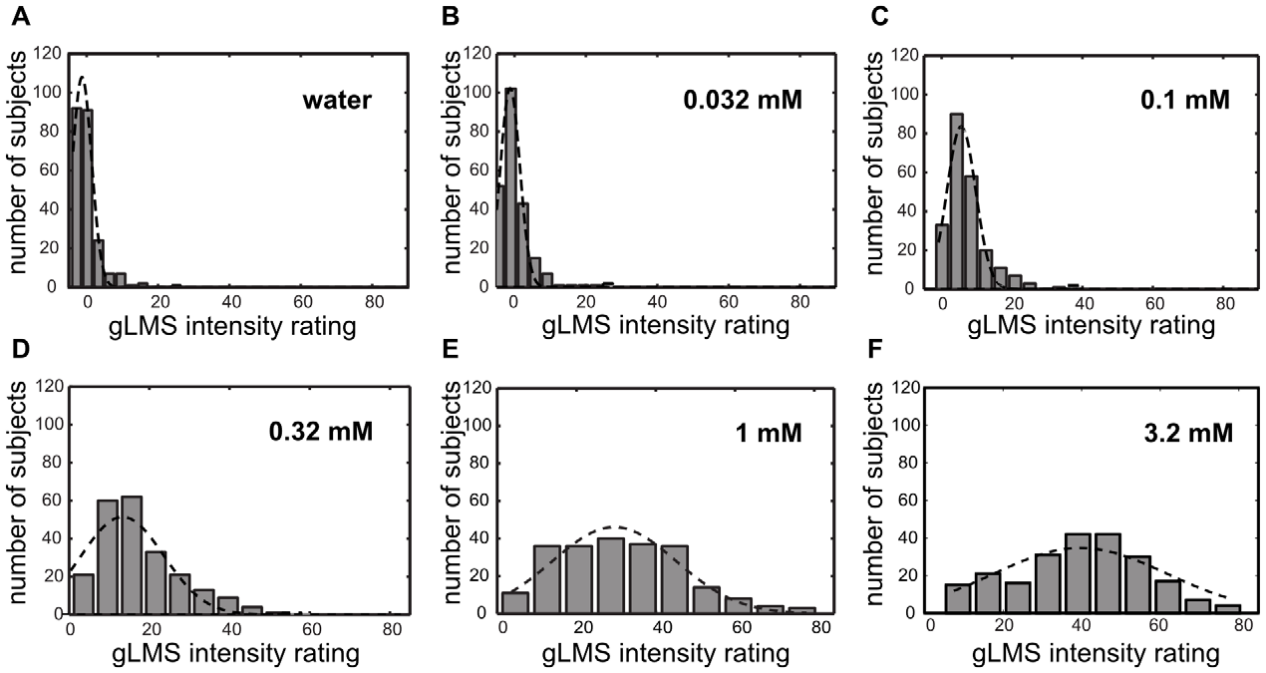
Inter-subject variability of PROP taste intensities. Distributions of gLMS intensity ratings (average of 3 measurements) for solutions containing 0.032, 0.1, 0.32, 1 and 3.2 mM PROP as well as pure water (panel a). Gaussians fits to the data are shown as dashed lines.

Determinations of generalized labeled magnitude (gLMS) intensity ratings were performed in triplicate. Figure 2 shows distributions for the intensity ratings for test solutions containing 0.032, 0.1, 0.32, 1 and 3.2 mM PROP respectively as well as for the control sample containing only water.

### Effect of gender, age, body mass index and ethnicity on PROP detection threshold

**Figure 3:**
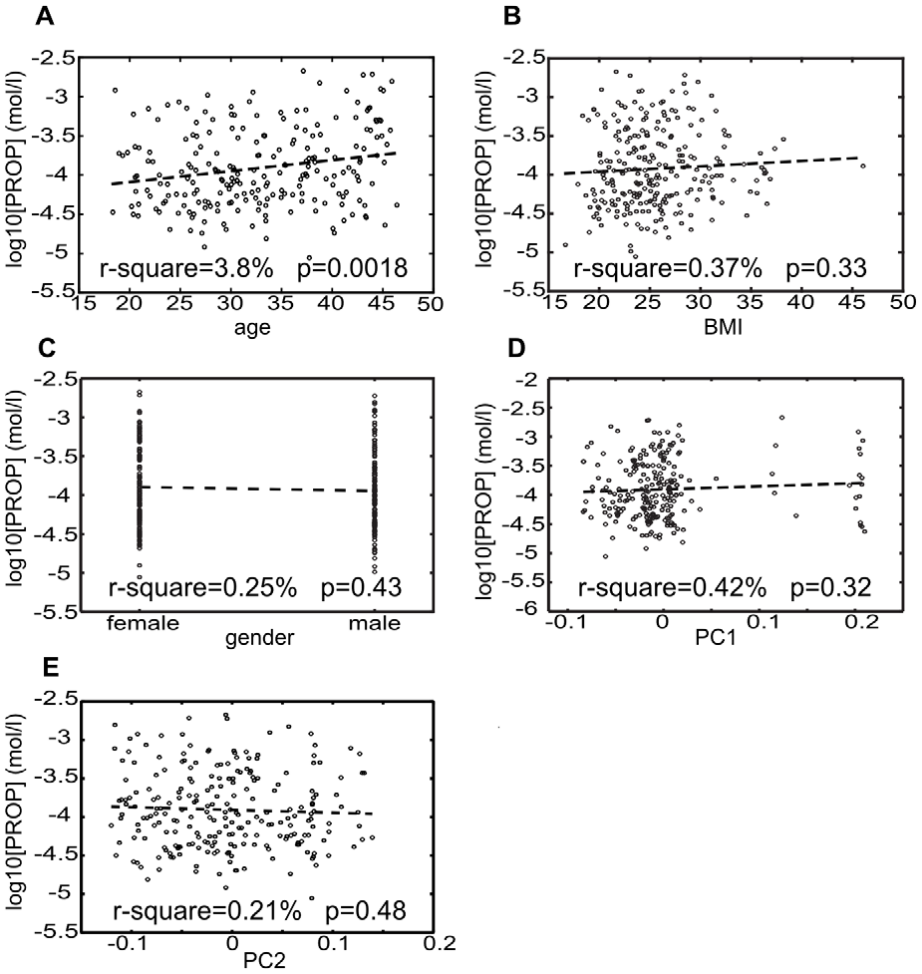
Influence of demographics on PROP detection threshold. Dependence of PROP detection thresholds on age (a), body mass index (b), gender (c) and principal components 1 and 2 (d,e) of a genetic ancestry analysis. A linear regression line and the corresponding r-square and p-values are indicated on each plot.

As part of the preparation for the GWAS analysis we assessed how the PROP detection threshold is affected by the key demographic parameters gender, age, body mass index and ethnicity. To assess the effect of ethnicity we performed linear regression against the first two principal components from a principal-component analysis of the entire genotyping data set. These principal components have been shown to be highly effective in describing and correcting for ethnic origin [23], [24]. The results, shown in figure 3, indicate that these demographic parameters have only a very modest influence on PROP detection thresholds. Among them, only age (Figure 3a) showed a statistically significant, positive association with detection threshold (p = 0.0018, r-square = 3.8%).

### GWAS of PROP detection threshold

The results of genome-wide association studies are commonly displayed in “Manhattan plots”. These plots show, for each of the ∼800,000 probed SNPs, the strength of the statistical association between the subjects' genotype and phenotype. The strength of the association is plotted as the negative decadic logarithm (-log_10_ (*p*)) of the probability *p* that an association of the observed strength could have occurred by chance.

**Figure 4:**
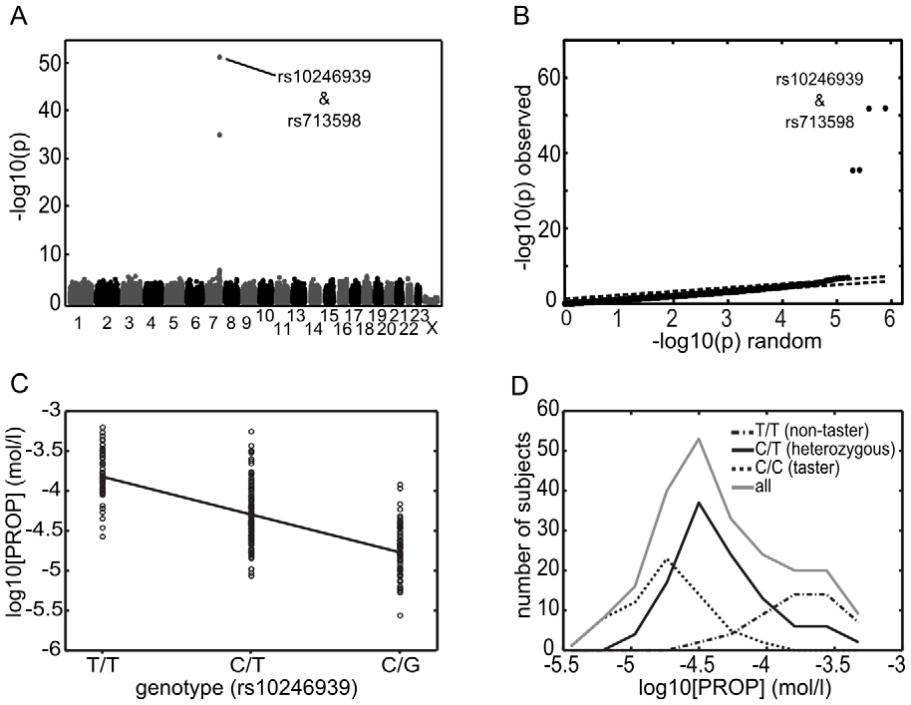
GWAS on PROP detection threshold. Results of the GWAS analysis for PROP detection threshold. The Manhattan plot (a) shows for each of ∼800,000 SNPs the –log_10_ (p) value of linear regression between the genotype at that SNP and the PROP detection threshold. The x-axis indicates the location of each SNP according to chromosome and position within the chromosome. Note the cluster of highly significant SNPs located on chromosome 7. The strongest associations correspond to TAS2R38 SNPs rs713598 and rs10246939. Due to their close proximity in the TAS2R38 gene and their similar p-values the markers for these two top SNPs overlap in this figure. The QQ-plot (b) is a scatter plot in which all ∼800,000 SNPs are ranked by their observed p-value (y-axis) and plotted against a rank-sorted p-value distribution (x-axis) representing the null hypothesis of random association. The very clear off-diagonal location of the top SNPs rs713598 and rs10246939 indicate the high statistical significance of the genotype-phenotype association. Panel (c) shows a linear regression of the subject's PROP detection threshold vs. their genotype at SNP rs10246939. The PROP detection threshold is represented on a logarithmic scale. Panel (d) shows distributions of the PROP detection thresholds according to subjects' genotype, the vertical axis indicates the number of subjects whose detection thresholds fall in the corresponding concentration bins.

Figure 4a shows the Manhattan plot for the GWAS on the detection threshold of PROP. The plot shows that the genotype for a small group of single-nucleotide polymorphisms on chromosome 7 is strongly associated with the PROP detection threshold. In perfect agreement with the results reported by Kim and colleagues [19] the strongest associations are to SNPs rs713598 (C->G) and rs10246939(C->T) which cause the well-known amino-acid substitutions (P->A at aa49 and V-> I at aa296) in the bitter taste receptor TAS2R38.

The profound statistical strength of the association (p  =  1.2×10^−52^ and 1.5×10^−52^ respectively for the top two SNPs) is a reflection of the near Mendelian inheritance of the PROP taster phenotype.

Figure 4b shows the QQ- plot for the GWAS of the PROP detection threshold. The vast majority of SNPs follow the p-value distribution expected for random association – *i.e.* they lie on the plot's diagonal. Only a small handful of SNPs, those located within or near to the TAS2R38 gene, show a significant deviation from this diagonal. Therefore the plot indicates that phenotype distributions conform to statistical expectation and that population structure effects, if they existed, have been corrected efficiently.

### Effect of TAS2R38 genotype on PROP detection threshold

The regression plot (figure 4c) of subjects' PROP detection thresholds *vs.* the subjects' genotypes at SNP rs10246939 underlines the strength of the association between TAS2R38 genotype and PROP detection threshold. The genotype at this SNP accounts for approximately half (49%) of the observed variability in detection thresholds. Distribution of PROP detection thresholds according to SNP rs10246939 genotype (figure 4d) show that, as a population, subjects who carry two copies of the C allele are least sensitive to PROP (log-averaged threshold  =  0.37 mM) followed by subjects who carry one C and one T allele (0.113 mM) and by subjects with two T alleles (0.047 mM).

### Optimal choice of taste phenotype representation

The input phenotype for the GWAS on the PROP detection threshold was the average of the two log-transformed replicate measurements of the detection threshold. This specific treatment of the threshold data was chosen to take into account the well-known, near-logarithmic response of humans to taste stimuli. Still, the decision for this particular treatment of the data represented a discreet choice. To investigate, if this choice was indeed optimal, or if there may be different phenotype representations that would increase the statistical power of the sensory data, we tested the performance of four other parameter representations in separate GWA analyses: i. the average of the raw (*i.e.* non log-transformed) detection threshold, ii. the log-transform of the average of the raw detection thresholds, iii. a scenario where the two observations of the detection threshold were both log-transformed but then treated as if they were independent observations on two separate, but genetically identical, subjects and finally iv. classification of subjects into tasters and non-tasters with the resulting binary phenotype analyzed by logistic regression (see next section). In this context the p-value of association serves as a measure of the statistical power provided by a particular phenotype representation. The lower the p-value achieved by a particular phenotype representation in this analysis, the greater is the statistical power afforded by it and the better it is suited to identify associated SNPs in future studies.

**Figure 5:**
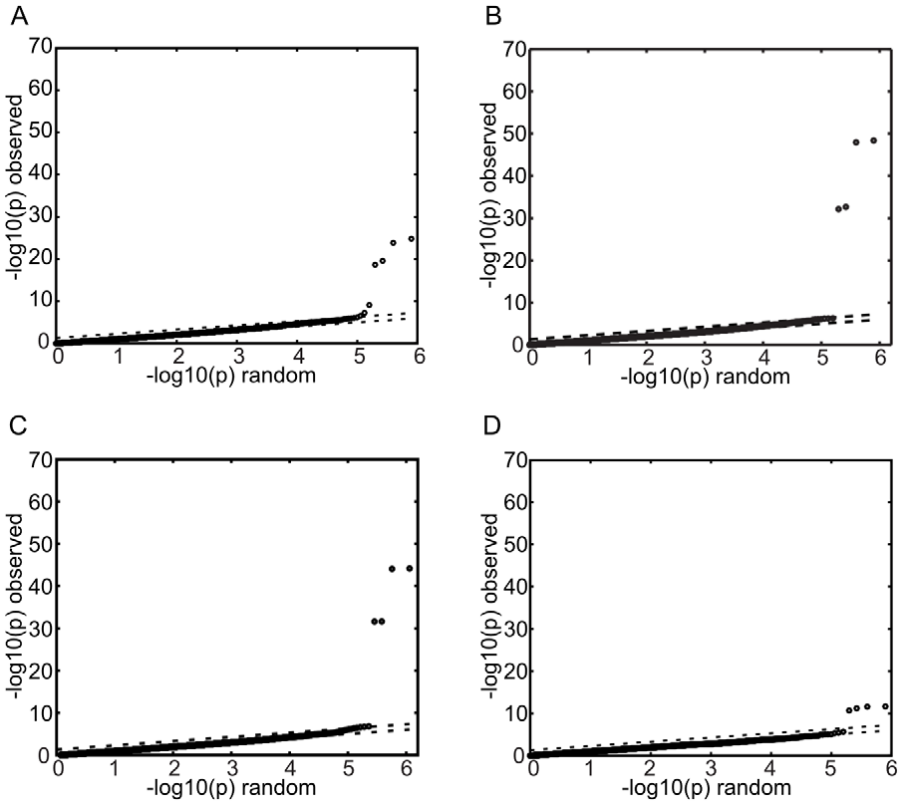
Effect of phenotype representation on GWAS signal. QQ-plots resulting from the GWAS analysis using different representations of the PROP detection threshold. (a) average of the raw (*i.e.* non-log transformed) detection thresholds (b) averaged, then log-transformed, thresholds (c) repeat measurements treated as independent observations. Panel (d) shows the QQ-plot resulting from a logistic-regression GWAS after classifying subjects into tasters and non-tasters based on a PROP detection threshold cutoff of 0.2mM.

Figure 5 shows the QQ- plots resulting from this analysis. The top four SNPs in each of the plots are identical to those identified by the initial GWA analysis described above. In other words, choosing a different representation for the phenotype parameter did not change the SNPs that were associated with the phenotype. But, the choice had a very dramatic impact on the strength of the association. For example, the average of the raw (*i.e.* non-log-transformed) detection thresholds (Figure 5a), which might *a priori* seem to be a reasonable choice for the input phenotype, performs rather poorly (p_min_ = 1.4×10^−25^). Also, averaging the detection thresholds before log-transformation noticeably weakens the association (p_min_ =  4.4×10^−49^) (Figure 5b).

The result for the third option, *i.e.* the treatment of the replicate threshold measurements as independent observations, is rather interesting. While this choice of parameter representation results in a very strong association (p_min_ = 6.6×10^−69^) prior to genomic control, the uncorrected QQ-plot (not shown) indicates substantial non-specific inflation of p-values for all SNPs. After genomic control [25] correction that adjusts for such non-specific inflation the p-value of the top SNP decreases to 7.3×10^−45^ (Figure 5c) well below the p-value obtained with the log-averaged representation of the detection threshold. The cause of these p-value inflations appears to be that, despite the large intra-subject variability, the repeat measurements of the detection thresholds from the same subjects are sufficiently correlated to violate the independence assumption of normal linear regression.

### Linear regression vs. logistic regression based on taster/non-taster classification

To compare the relative power of the linear-regression-based GWAS described above to a GWAS based on classifying subjects into tasters and non-tasters we performed the following analysis. We classified subjects into tasters and non-tasters using the standard detection threshold cutoff of 0.2mM [2]. We then performed a GWAS using logistic regression for the binary taster/non-taster phenotype. To ensure consistency with the linear-regression GWAS we used the same covariates and genomic corrections in both analyses.

While the logistic approach was able to identify the TAS2R38 SNPs, the association is dramatically weakened (p_min_ = 2.54×10^−12^) compared to the linear regression analysis (p_min_ =  1.2×10^−52^). To ensure this weak association was not due to the choice of the cutoff concentration used for taster-vs.-non-taster classification we repeated the analysis by varying the cutoff from 0.1mM to 0.3mM in 0.025 mM steps. Still this “optimization” of the cutoff value could not enhance the association (p_min_ = 2.25×10^−12^ with a 0.175 mM cutoff). These results illustrate the very substantial loss of statistical power that results when noisy quantitative phenotypes are converted into binary phenotypes.

### Trade-offs between phenotype quality and panel size

The results described in the previous section answer the question of how best to treat replicated measurements of detection thresholds for use as input phenotypes in a GWAS. But, is the collection of repeat measurements useful? More precisely, is it better to perform replicate measurements on a smaller number of subjects, thus increasing phenotype quality, or, is it better to perform just a single measurement on as many subjects as possible? This latter option may, for example, be attractive when phenotyping is performed as an add-on to an existing genotype-phenotype association study. In such a scenario recruitment and genotyping costs are not a factor and the only question is how to best use the available phenotyping resources. To address this question we performed a GWAS analysis on all 225 subjects of our study but used only the first measurement of the detection threshold. We then compared the result from that GWAS to results from GWASs performed by randomly selecting sub-panels of half the number of subjects (i.e.113) and using the average of the two detection threshold replicates as the phenotype. To avoid sampling bias, we repeated the sub-panel selection 1000 times and averaged the results. The comparison shows that the GWAS using a single threshold determination on 225 subjects (p_min_ = 9.4×10^−41^) clearly outperformed that on the log-averaged detection threshold of 113 subjects (log-averaged p_min_ = 8×10^−27^). These results are fully in line with the general rule that replicate measurements add less power to an association study than the addition of an equivalent number of new subjects.

### GWAS on supra-threshold intensity ratings

**Figure 6:**
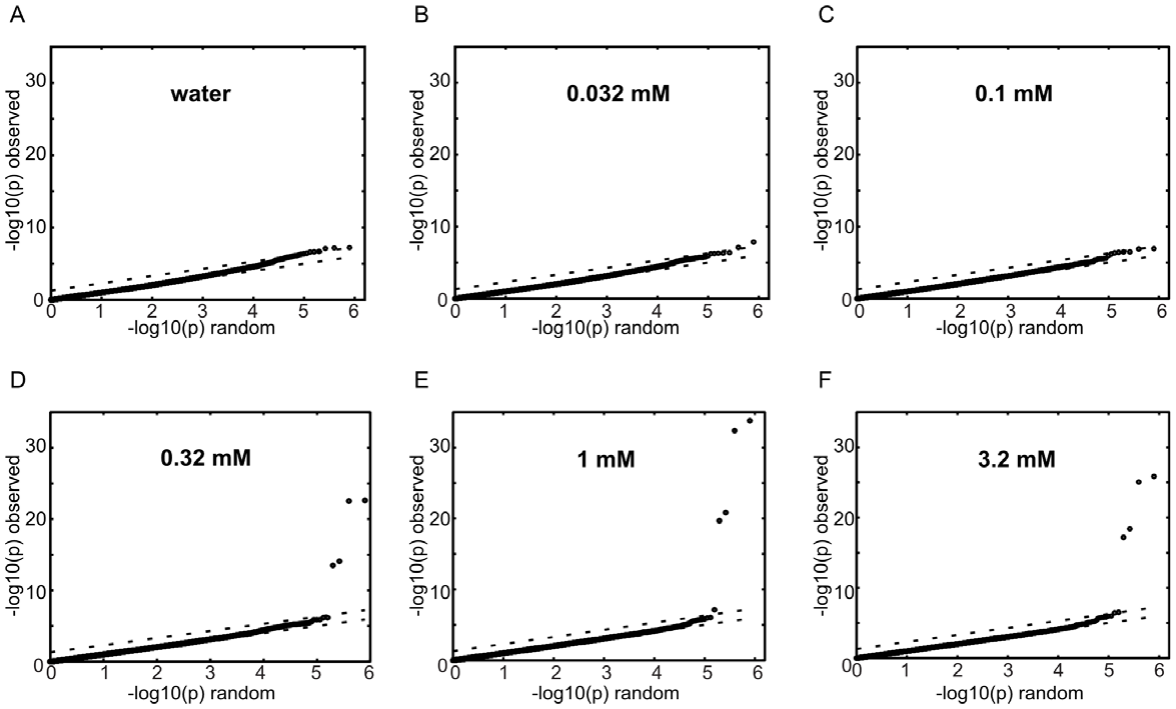
Effect of PROP concentration on strength of GWAS signal. QQ-plots for the GWAS analysis of the gLMS intensity ratings of a water control (a) and PROP solutions at 0.032 mM (b), 0.1 mM (c), 0.32 mM (d), 1 mM (e) and 3.2mM (f).

To compare the relative power of objective taste sensitivity measurements (see above) to measurements obtained by subjective methods we performed GWAS on the supra-threshold intensity ratings obtained with the gLMS method. Specifically, we performed separate GWAS's for the intensity ratings for each of the five concentrations of PROP included in our gLMS tests as well as for the water control. As input phenotype we used the average of the three intensity ratings for each of the concentrations (Figure 2). As the gLMS scale used to record intensity ratings is *de facto* a log-based scale we did not log-transform the intensity ratings. The QQ-plots from the GWA analyses are shown in figure 6. As might be expected, the gLMS ratings for water and for the two PROP concentrations (0.032 and 0.1 mM) that fall below the average detection threshold (0.12mM) of the panelists did not produce genome-wide significant associations. By contrast, the three concentrations above the average detection threshold produce clean and robust associations. With a p_min_ of 1.6×10^−34^ and an r-square of 38% the gLMS intensity rating for the 1mM PROP solution turns out to be nearly as powerful a phenotype as the detection threshold measured by the staircase method (see above). For each of the three concentrations where genome-wide significant associations were obtained the two most strongly associated SNPs are the same SNPs in the TAS2R38 receptor (rs713598 and rs10246939) that were identified in the GWAS with the PROP detection threshold.

**Figure 7:**
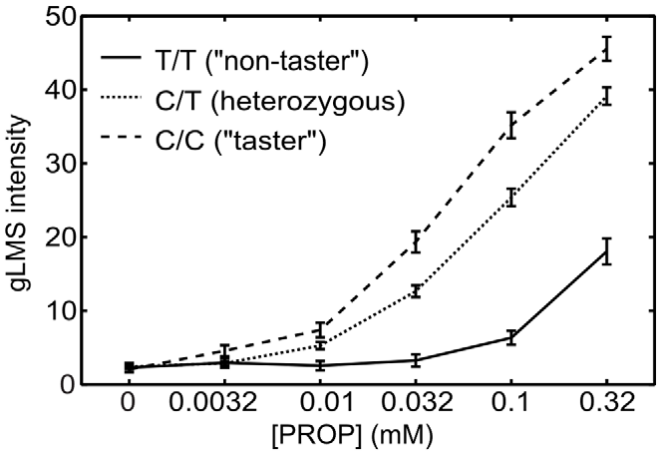
PROP taste intensity as function of TAS2R38 genotype: Average PROP intensity ratings as a function of PROP concentration shown according to subjects' genotype at TAS2R38 SNP rs10246939. Error bars show the standard error of the ratings.

Figure 7 shows the average gLMS response curves according to TAS2R38 genotype. Subjects carrying two copies of the non-taster version of TAS2R38 give the lowest intensity ratings across all sample concentrations while subjects with two taster versions give the highest ratings and heterozygotes display an intermediary phenotype. Note that subjects who carry two copies of the non-taster allele rate PROP solutions of concentrations below that group's average detection threshold (0.37mM) with the same intensity as they rate water. This result indicates the consistency between our threshold and intensity rating data and also indicates that gLMS intensity rating data may be used to derive -indirectly- detection threshold information.

To evaluate the usefulness of phenotyping strategies that integrate all six data points from an individual's gLMS curve into a single parameter we calculated an “area-under-the-curve” phenotype (i.e. the sum of the six gLMS ratings that make up an individual's gLMS curve). It could be reasoned that this phenotype effectively pools the signal across all six sample concentrations and might therefore increase the strength of the association signal. At the same time, not all concentrations add an equal amount of signal, but all of them add noise. Along this line of thinking pooling of the signal across different concentrations might actually lead to a loss of power in the phenotype. The results of a GWAS with the area-under-the-curve phenotype show that, while this phenotype also correctly identifies rs713598 and rs10246939 as the top SNPs the overall strength of the association p_min_ = 8.2×10^−28^ is weaker than for the straight gLMS rating of the 1mM solution alone (see above) indicating that pooling of intensity ratings across different concentrations does not necessarily result in a increased association signal.

### Relative power of detection and minimal number of subjects for achieving genome-wide significance

**Figure 8:**
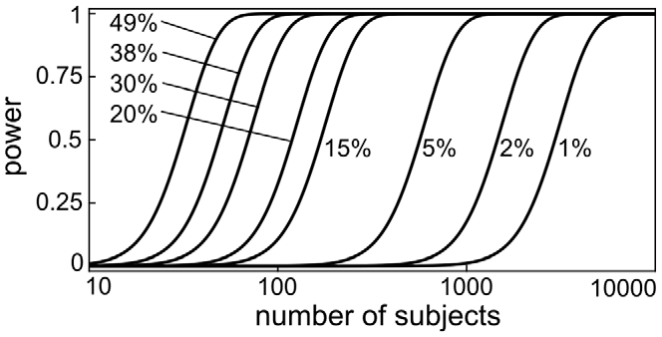
Power-to-detect curves . Curves were calculated for r-square values of 49% corresponding to the variance in PROP detection threshold explained by the top TAS2R38 SNP and 38% corresponding to the explained variance for gLMS intensity rating for a 1mM solution of PROP. Calculations were based on a genome-wide significance threshold of p = 5×10^−8^. For comparison power to detect curves for 30, 20, 15, 5, 2, and 1% variance explained are also shown.

To compare the relative power of detection-threshold and supra-threshold measurements as input phenotypes for GWA studies we calculated power-to-detect curves for the two phenotypes. For these calculations we used a p-value cutoff of 5×10^−8^ to denote genome-wide significance and r-square values for PROP detection thresholds (49%) and the supra-threshold intensity ratings of the 1mM PROP solution (38%) as determined in our study (see above). The resulting power-to-detect curves (figure 8) show the exceptional power of both the threshold and intensity rating parameters. For the detection threshold, well below 100 subjects are sufficient to obtain a robust association (p-value <5×10^−8^) with greater than 90% probability. To provide an indication for the number of subjects that would have been required to detect additional genetic factors with weaker influence on PROP sensitivity we also computed power to detect curves for a number of smaller r-square values. As can be seen from these curves, our panel size of 225 subjects would not be sufficient to detect genetic factors that explain less than ∼15% of overall phenotype variance.

## Discussion

### Psychophysics Measurements

The results of the psychophysics measurements reported here, both in their average values and in the observed distribution of values are in agreement with recent observations by others [13], [21], [22]. One feature that distinguishes our study from most recent studies is the replication of the psychophysics test on the same panel of subjects within a relatively brief period of just two weeks. Over this period, threshold determination was performed in duplicate and supra-threshold intensity curves were recorded in triplicate.

The intra-subject variability between those repeat measurements is substantial – both for the detection thresholds and for the supra-threshold intensity ratings. In the case of the detection thresholds the average difference between the two measurements is 0.32 log_10_ units, which corresponds to a greater than two-fold change in the detection threshold. And, for 17% of subjects the variability between replicate measurements was >0.62 log_10_ units corresponding to a greater than four-fold change in detection threshold. As a consequence, some subjects that would have been classified as tasters based on the first measurement later displayed detection thresholds that would classify them as non-tasters and vice versa.

This intra-subject variation in phenotype far exceeds the nominal resolution of the stair-case method that was used to measure this phenotype. Therefore it is unlikely that this variation is simply the result of measurement error. Instead, other factors which are not easily controlled do appear to have a substantial effect on day-to-day variations in taste sensitivity.

The intra-subject variability we observed in our study over a rather short period of just a few days is comparable in magnitude to the intra-subject variability reported in studies with substantially longer time periods of weeks to months between repeat measurements [26]–[28].

Together the data underline that neither a subject's detection threshold nor its taster/non-taster status should be viewed as a single, fixed parameter that can be ascertained in a single measurement session. Instead, a subject's detection threshold appears to be a dynamic parameter that is subject to substantial variation over the period of just a few days.

The same caveat applies to the supra-threshold intensity ratings. In fact, intra-subject variability of the supra-threshold ratings was even greater than that of the detection thresholds. Intensity ratings involve a larger degree of subjectivity than do the threshold determinations via the staircase method. Correspondingly, the gLMS scores were somewhat less powerful in identifying TAS2R38-PROP association in our GWA studies. Still, the GWA studies based on the gLMS scores easily identified the correct SNPs. And gLMS scores do have key advantages that, despite this reduced power, may make them attractive phenotyping parameters for future GWA studies in the field of psychophysics. First, after an initial training of participants, gLMS intensity curves can be recorded much faster and with lower personnel effort than detection thresholds. In cases where the costs of genotyping and subject recruitment are not limiting, but testing time is at a premium, gLMS intensity measurements may be particularly attractive. Such a situation may be encountered, for example, when psychophysics measurements are performed as an “add on” to another study. Also, gLMS intensity curves provide both a rough estimate of a compound's detection threshold as well as supra-threshold intensity ratings. In cases where variations in detection thresholds and variations in the perceived intensity of supra-threshold stimuli are driven by two distinct genetic variations, gLMS intensity curves may then allow the identification of both of these genetic variations.

### Other genetic factors of PROP taste perception

Past studies have suggested that genetic factors, other than TAS2R38, may also play a role in driving the observed natural variation in the bitter taste perception of PTC and PROP. In table 1 we show the list of all 34 SNPs that generated genome-wide significant or “suggestive” p-values (<10^−5^) in the GWAS on the PROP detection threshold phenotype. Only four of these SNPs have genome-wide significant associations (p<5×10^−8^) and all four of these SNPs are located within the TAS2R38 gene itself or within its immediate vicinity. Incidentally the same four SNPs (rs713598, rs10246939, rs4726481 and rs1726866) were the only SNPs that achieved genome-wide significance for any of our studied phenotype representation.

**Table 1:** PROP detection threshold-associated SNPs with p<10^−5^.

SNP_name	Chr	pos	gene	MAF†	p-value
rs713598	7	141319814	TAS2R38*	48%	1.21×10^−52^
rs10246939	7	141319073	TAS2R38*	46%	1.49×10^−52^
rs4726481	7	141314872	TAS2R38*	47%	3.04×10^−36^
rs1726866	7	141319174	TAS2R38*	41%	3.95×10^−36^
rs17162635	7	141324432	TAS2R38*	10%	1.17×10^−07^
rs11767947	7	141259090	OR9A4*	23%	1.53×10^−07^
rs12539499	7	141276736	CLEC5A*	23%	1.90×10^−07^
rs11767119	7	141258585	CLEC5A*	23%	2.55×10^−07^
rs6976028	7	141259186	OR9A4*	24%	3.37×10^−07^
rs4725559	7	140934121	AGK*	37%	5.38×10^−07^
rs4726463	7	141013108	KIAA1147*	36%	6.10×10^−07^
rs11762634	7	141302956	TAS2R38*	20%	8.11×10^−07^
rs10464444	7	141178656	LOC136242*	26%	1.03×10^−06^
rs11773340	7	140963303	AGK*	39%	2.44×10^−06^
rs12944462	17	38391027	RUNDC1	39%	2.47×10^−06^
rs1374825	3	141047747	CLSTN2	28%	2.60×10^−06^
rs3774700	3	62291434	C3orf14	12%	3.23×10^−06^
rs2273549	11	33036156	TCP11L1	13%	4.26×10^−06^
rs12365818	11	33059853	CSTF3	12%	4.63×10^−06^
rs2570407	7	141301361	CLEC5A*	22%	5.04×10^−06^
rs455055	17	38421483	VAT1	38%	5.54×10^−06^
rs12703413	7	141193316	LOC136242*	23%	5.87×10^−06^
rs3912601	3	73494377	PDZRN3	39%	5.94×10^06^
rs1052990	7	115935606	CAV2	34%	6.00×10^−06^
rs7717550	5	151856950	#	14%	6.83×10^−06^
rs10261374	7	141045057	KIAA1147*	36%	6.94×10^−06^
rs2851758	18	23570812	#	32%	7.30×10^−06^
rs9978374	21	14741592	SAMSN1	44%	7.36×10^−06^
rs7218454	17	38359611	AARSD1	40%	7.41×10^−06^
rs7717092	5	151856655	NMUR2	13%	8.08×10^−06^
rs13231650	7	141045411	KIAA1147*	40%	8.27×10^−06^
rs12373190	18	23596043	#	17%	8.52×10^−06^
rs7969452	12	18578000	PIK3C2G	21%	9.10×10^−06^
rs3768812	2	210264021	MAP2	48%	9.37×10^−06^

*These SNPs are located within or within the immediate vicinity of the TAS2R38 gene.

# These SNPs are not located within the vicinity of a known gene (>100kb form the closest annotated gene)

†Minor Allele Frequency.

An additional 14 SNPs are located very near TAS2R38. To test, if these SNPs are simply in linkage disequilibrium with the causal TAS2R38 SNPs, or if they make an independent functional contribution, we corrected each subject's phenotype for their genotype at the top TAS2R38 SNP and then used this corrected phenotype in an additional GWAS. In this second GWAS the p-value for these 14 SNPs decreased to well below statistical significance indicating that they do not make an independent contribution to the PROP detection phenotype.

The remaining 16 SNPs with suggestive p-values of <10^−5^ are distributed throughout the genome with 13 of them within less than 100kb of an annotated gene. None of these genes suggested a mechanistic link to taste perception.

Previous studies had specifically implicated loci on chromosome 16p [29] and chromosome 5p15 [30]. With 10165 and 6330 quality controlled SNPs respectively these two chromosomal regions are well covered by the SNP chip used in our experiment. We generated local Manhattan and qq-plots for both of these regions (data not shown). In both cases the distribution of p-values is fully consistent with purely random associations.

On the p-arm of chromosome 16 the minimal p-value of 2.8×10^−4^ is observed for rs9933117 which is located in an intron of *XYLT1,* a xylosyltransferase gene. After Bonferroni correction for the large number of SNPs in this region the p-value is non-significant (p_corr_  =  1). A similar situation is observed for the 5p15 region. Here the minimum p-value of 3.6×10^−4^ is observed for SNP rs1395093. This SNP falls into a gene-free region and after Bonferroni correction the p-value also drops to a non-significant level (p_corr_  =  1).

We further find that variations in both detection threshold and supra-threshold intensities are dominated by the same genetic variations in the TAS2R38 receptor. These observations are entirely consistent with recent results from *in vitro* experiments on functionalized bitter receptors [31] that indicate that both PROP and PTC activate only TAS2R38 and no other bitter taste receptor.

At the same time we like to make clear that failure of our study to detect additional genetic variants that impact PROP perception in no way precludes the existence of such genetic variants. It is very well possible that more powerful studies (*e.g.* studies on larger panels or different cohorts) may identify such associations in the future. Still, we can use the data from the current study to get a rough idea about the likely upper limit for the impact that such genetic variants could have. Using power-to-detect calculations we can see that with a panel of 225 subjects and a genome-wide-significance cutoff of log(p)  =  5×10^−8^ a genetic variation that explains more than 11.5% of overall phenotype variance would have been detected in our GWAS with greater than 50% probability. In other words, failure to find additional genetic factors in our study makes the existence of multiple genetic factors with substantial impact on PROP taste perception (r-square >> 11.5%) seem unlikely.

Correspondingly, we can conclude that our current data set is not powerful enough to detect genetic variations with subtle impacts (r-square <11.5%) on PROP perception via the hypothesis-free GWAS approach. Still, this data set may enable other researchers to study such subtle genotype-PROP-taste associations using a target-gene approach. The complete set of the association p-values for the PROP detection threshold phenotype for all genotyped SNPs is included in Supporting Information S1. The SNPs contained in this data set have passed our quality control criteria, were calculated based on phenotype scores corrected for covariates and have undergone genomic control as described in the methods section.

### Effect of genetic ancestry on PROP taste perception

Given the very substantial differences in the frequency of the relevant TAS2R38 genotypes in different ethnic groups (see HapMap data base [32]) one might have expected an association between genetic ancestry principal components and PROP taste sensitivity. But, no such association was observed. We attribute this lack of association to the population structure of our panel. The ancestry principal component plot of our panel (data not shown) indicates a continuous admixture of genotypes from different ethnic origins. As a result of this admixture the link between the specific TAS2R38 genotype, which drives PROP taste sensitivity, and the overall genetic ancestry, which represents ethnicity, will be weakened – apparently to the point where a statistical association can no longer be observed.

### Non-genetic factors influencing PROP taste perception

Approximately 50% of the observed variation in PROP detection thresholds is accounted for by genetic variation in the TAS2R38 gene. From replicate measurements we can estimate that the measurement error accounts for another 20% of the observed phenotype variability. This leaves approximately 30% of the phenotypic variation we observed to be explained by other factors. Consistent with reports in the literature [33]–[35] neither gender nor body mass index explain a significant portion of these remaining 30% of variation. Even age (Figure 3a), which is known to influence both overall [34] and PROP-specific [26] taste sensitivity explains only 3.8% of the overall variation in PROP detection thresholds.

### Conclusions for GWAS studies on other chemosensory phenotypes

The results of the current study, together with the fact that common genetic variations with strong impact on sensory phenotype seem to exist [8], [9] beyond the PROP-TAS2R38 association, indicate that GWAS studies have the potential to yield important discoveries in the field of human chemosensory perception. Specifically, given a sufficiently strong influence of the genotype on the sensory phenotype, the large, and as we think inherent, uncertainty in the measurement of chemosensory phenotypes will not pose a fundamental obstacle to the success of the GWAS strategy.

Our study shows that careful selection of the measured phenotype and equally careful processing of the data can significantly boost the power of an association signal. The optimal phenotpying strategy will depend critically on a study's setting. For example, in our study detection thresholds determined with the staircase method were clearly the most powerful phenotype for identifying the PROP-TAS2R38 association. However, due to its complexity the staircase protocol required both a greater time commitment from panelists and a substantially greater personnel effort than the measurement of gLMS taste intensity curves. Taking this difference in effort into account, the gLMS phenotype, which is slightly less powerful than the detection threshold phenotype, is clearly the more efficient phenotyping method. Therefore, if phenotyping is carried out on a large, already genotyped cohort, the simpler gLMS approach would appear to be highly attractive. In fact, recent GWASs on quinine taste perception [8] and specific anosmias [9] have shown that even extremely streamlined phenotyping approaches can be successful as long as this simplicity in the phenotyping method enables access to very large, already-genotyped subject panels.

Below are 5 points of concrete advice that summarize the findings from our study. Taking these 5 points into account in the design of future chemosensory GWAS studies should help boost the genotype-phenotype association signal and with it boost the chance to identify genome-wide significant associations:

1. Conversion of a continuous phenotype (*e.g*. detection threshold) into a binary phenotype (*e.g*. taster/non-taster status) generally leads to a substantial loss of statistical power and should be avoided. While this loss of power is well-known and well-understood from a statistical perspective, conversion of taste thresholds into taster/non-taster status continues to be such a common practice in the taste field that we feel compelled to point out the resulting loss of statistical power in this context.
2. Investing phenotyping resources into the testing of additional panelists adds substantially more power to an association signal than replication measurements on existing panelists. If information from replicate measurements is desirable (e.g. to obtain information on phenotype accuracy or on the variation of a phenotype over time), we recommend to perform such replicate measurements only on a subset of the panelists. Typically a rather modest number of subjects will already be sufficient to get a reliable estimate of intra-subject variability so that the bulk of the phenotyping effort can be dedicated to the inclusion of additional subjects.
3. Detection thresholds measured via the staircase method are a more powerful phenotype than suprathreshold intensity ratings obtained with the gLMS approach. Threshold measurements represent an attractive phenotyping strategy when panel size is limited.
4. Determination of suprathreshold intensity ratings using the gLMS approach is a more cost effective phenotyping strategy. Use gLMS-based approaches for phenotyping when large, already genotyped subject panels are available.
5. Detection threshold data should be log-transformed and then -if replicate measurements were obtained- averaged to generate the input phenotype for the GWAS. This log-transformation accounts for the log-linear relationship between taste stimulus and taste response, leads to normally-distributed residuals in genotype-phenotype regression during GWAS and ultimately boosts the power to find genotype-phenotype associations.

Given that taste perception of PROP bitterness follows the prototypical stimulus-response relationship (figure 7) found across much of psychophysics the above points will be directly transferable to a wide range of sensory phenotypes.

In conclusion, we have used the well-known association between variations in the TAS2R38 taste receptor gene and variations in taste perception of the bitter compound PROP to evaluate the relative power and efficiency of different chemosensory phenotyping strategies for genome-wide association studies (GWAS). The performance of the GWAS was surprisingly robust to the specific choice of data processing procedures and both detection threshold as well as supra-threshold intensity ratings reproduced the TAS2R38 association unequivocally. Still, careful choice of phenotyping method and parameter representation can provide a substantial boost in the strength of the genotype-phenotype associations. We anticipate that the lessons learned in this study will be valuable for future GWA studies on chemosensory phenotypes where associations between genotype and phenotype are less pronounced than is the case for TAS2R38 and PROP detection.

## Materials and Methods

### Ethics Statement

All procedures were approved by the Institutional Review Board of the Sírio Libanês Hospital, where tests were administered, and by the National Committee of Research Ethics at the Brazilian Ministry of Health (HSL 2007/25 Process no. 25000.114841/2007-17).

All subjects gave informed consent by signing a written informed consent statement.

### Participants

A group of 225 subjects was recruited from the general population of the São Paulo metropolitan area of Brazil. Subjects were aged 18-47 (mean 32.8). BMIs ranged from 16.6 to 46.1 (mean 25.5). In the panel 49% of subjects were male and 51% female. The Sao Paulo metropolitan area is known for its high degree of ethnic diversity and this diversity is reflected in the panel. Subjects were required not to smoke 3 hours prior to testing. A detailed breakdown of subject gender, age, BMI and smoking status is provided in table 2.

**Table 2:** Panel Demographics.

	male	female	total
**# subj.**	113	112	225
**BMI avg./min/max**	25.25/18.68/46.1	26.03/16.6/38.2	25.53/16.6/46.1
**<15**	0	0	0
**15–20**	8	7	15
**20–25**	55	48	103
**25–30**	40	34	74
**30–35**	6	16	22
**35–40**	3	7	10
**>40**	1	0	1
**Age avg./min/max**	30.98/18.34/45.89	34.85/20.38/46.39	32.81/18.34/46.39
**<20**	5	0	5
**20–25**	19	11	30
**25–30**	36	13	49
**30–35**	17	34	51
**35–40**	18	21	39
**>40**	18	33	51
**Smoker**	24	24	48
**Non-smoker**	89	88	177

### Genotyping

Genotyping services were outsourced to Expression Analysis Inc. (Durham, NC, USA). Briefly, genomic DNA was extracted from whole blood and genotyping was performed on the Illumina Human Omni-Quad1 platform following standard protocols. Genotype calling was performed with Beadstudio software (Illumina). Calls with a genotyping score below 0.2 were excluded from further analysis. Single nucleotide polymorphisms (SNPs) with a call rate below 90% and individuals with a call rate below 95% were also excluded.

Genotyping data was of high quality with an average call rate of 99.8% for all SNPs. 99.4% of SNPs had a call rate of greater than the cutoff value (95%) set for the rejection of individual SNPs. The average Q-score for all SNPs was 0.71 and for 99.6% of called SNPs the Q-score passed the cutoff (0.2) for inclusion.

### Determination of detection thresholds

Detection thresholds for PROP were determined via the staircase method [13], [14] with a four down, one up, five reversals protocol. The detection threshold was calculated as the average of the last four reversal concentrations. Solutions of PROP were prepared in milli-Q water. Dilution along 1/6^th^ log steps (equivalent to 1.468-fold dilutions) was used to generate 25 test solutions ranging in concentration from 3.2 mM to 0.32 microM. Based on the results of a preliminary PTC paper-strip test, the staircase procedure was started at a PROP concentration of 0.047 mM for PTC-tasters and 0.689 mM for non-tasters. Between samples subjects rinsed their mouth three times with milli-Q water. The staircase procedure was repeated after one week to obtain a second measurement.

### Determination of supra-threshold intensities

Supra-threshold taste intensities were determined using a general labeled-magnitude scale (gLMS) procedure [36]. Prior to the first tasting session subjects participated in a training session on the use of the gLMS. In each of the tasting sessions subjects were presented with solutions of PROP dissolved in milli-Q water at five concentrations (3.2 mM, 1mM, 0.32 mM, 0.1 mM and 0.032 mM.) The five samples, plus a control sample containing only water, were presented in random order. Sample presentation in random order, instead of presentation in order of increasing concentration, was chosen to eliminate bias in intensity ratings. The sample size was 20 ml. Subjects tasted each sample separately using a sip-and-spit protocol and rated the intensity of the taste sensation on a vertical general labeled magnitude scale. Between samples subjects rinsed their mouth three times with milli-Q water. Each subject performed this gLMS test 3 times. The tests were spaced out over a period of two weeks.

### Preparation of phenotype data

The two repeats of the detection threshold determined by the staircase method were log-transformed and then averaged. For comparison purposes we also calculated other representations of the detection threshold (see results section for more details).

For the gLMS data intensity ratings for a given PROP concentrations were averaged for each subject and the averaged rating for a specific concentration was used as the input phenotype for GWA analysis.

### Genotype-phenotype associations

Genotype-phenotype association analysis was performed with in-house Matlab (The MathWorks, Inc., Natick, MA, USA) code optimized for GWAS scenarios.

Population stratification and relatedness was assessed using the ancestry principal components as previously described [23], [24], [37]. Input phenotypes were corrected for essential covariates via linear regression. We used age, sex, body mass index and the first 10 principal components of the ancestry analysis as covariates. The residuals from this regression, *i.e.* the corrected phenotype parameters were then regressed against SNP allele dosage using a linear, additive model.

Logistic regression was performed using the same set of covariates. To determine the binary phenotype for logistic regression, subjects were classified into PROP-tasters and non-tasters according to their log-averaged detection threshold determined via the staircase method.

Genomic control [25] was applied to the genome-wide p-values to detect and correct for p-value inflation. If not noted otherwise, the determined p-value inflation factors (lambda) indicated no significant inflation and confirmed that possible population stratification was sufficiently corrected via inclusion of the top 10 ancestry principal components as covariates.

A complete GWAS including, phenotype correction and genomic control required less than 3 minutes of CPU time on an 8 processor 2.3 GHz Xenon-based computer with 8 GB of RAM running a RedHat implementation of Linux version 2.6.

### Power-to-detect curves

Power to detect curves, for details see [38], [39], were calculated using the standard formula 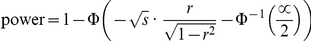
where r^2^ is the fraction of phenotypic variance explained by a SNP,  the normal cumulative distribution function, s the number of subjects and α the significance threshold (set to 5×10^−8^ for a GWAS scenario).

### Calculation of area under the curve phenotype from gLMS data

To calculate the area-under-the-curve phenotype from our gLMS data we averaged the three repeat measurements for each concentration and then added those average scores across the 6 measured samples (i.e. 5 PROP concentrations plus water) to obtain a representation of the “area” under the taste-intensity-vs.-concentration curve. See figure 7 for an example of such a curve.

## Supporting Information

Supporting Information S1 The file “genome_wide_p-value_table” contains a list of p-values for associations to the PROP detection threshold phenotype for ∼790′000 SNPs that were measured in our study. The SNPs have been quality controlled for Hardy-Weinberg Equilibrium p-value (>10^−5^), call rate (>95%), q-score (>0.2) and minor allele frequency (>5%) and have undergone correction for age, gender, BMI and genetic ancestry principal components as well as genomic-control. Individual data items are snp-identifier (e.g. rs number), chromosome number, position on chromosome, and p-value. The data is organized with one SNP per row and individual data items are separated by a semicolon. Chromosome number and position are based on the NCBI B36 build of the human genome.
(CSV)
