# Correction: Sensitivity of Genome-Wide-Association Signals to Phenotyping Strategy: The PROP-TAS2R38 Taste Association as a Benchmark

**DOI:** 10.1371/journal.pone.0122424

**Published:** 2015-03-30

**Authors:** The PLOS ONE Staff

The x-axis for Fig. 7 is incorrect by an order of magnitude. Please see the corrected Fig. 7 below.

**Fig 7:**
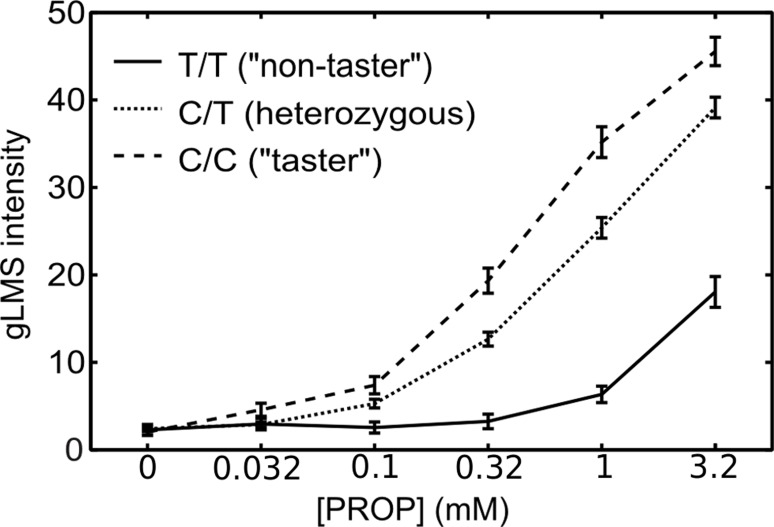
PROP taste intensity as function of TAS2R38 genotype: Average PROP intensity ratings as a function of PROP concentration shown according to subjects' genotype at TAS2R38 SNP rs10246939. Error bars show the standard error of the ratings.
